# Intelligent Sensory Pen for Aiding in the Diagnosis of Parkinson’s Disease from Dynamic Handwriting Analysis

**DOI:** 10.3390/s20205840

**Published:** 2020-10-15

**Authors:** Eugênio Peixoto Júnior, Italo L. D. Delmiro, Naercio Magaia, Fernanda M. Maia, Mohammad Mehedi Hassan, Victor Hugo C. Albuquerque, Giancarlo Fortino

**Affiliations:** 1 Graduate Program in Applied Informatics (PPGIA), University of Fortaleza, Fortaleza 60811-905, Ceará, Brazil; eugeniopeixotojr_@edu.unifor.br (E.P.J.); italo_lucena93@edu.unifor.br (I.L.D.D.); fernandamaia@unifor.br (F.M.M.); victor.albuquerque@ieee.org (V.H.C.A.); 2 LASIGE, Department of Computer Science, Faculty of Sciences, University of Lisbon, Campo Grande, 1749-016 Lisbon, Portugal; ndmagaia@fc.ul.pt; 3 Information Systems Department, College of Computer and Information Sciences, King Saud University, Riyadh 11543, Saudi Arabia; mmhassan@Ksu.edu.sa; 4 Department of Informatics, Modeling, Electronics, and Systems, University of Calabria, 87036 Rende, Italy

**Keywords:** Parkinson’s disease, machine learning, handwritten dynamics

## Abstract

In this paper, we propose a pen device capable of detecting specific features from dynamic handwriting tests for aiding on automatic Parkinson’s disease identification. The method used in this work uses machine learning to compare the raw signals from different sensors in the device coupled to a pen and extract relevant information such as tremors and hand acceleration to diagnose the patient clinically. Additionally, the datasets composed of raw signals from healthy and Parkinson’s disease patients acquired here are made available to further contribute to research related to this topic.

## 1. Introduction

Parkinson’s disease (PD) is a neurodegenerative disorder that affects an individual’s central nervous system, compromising its dopamine production, which causes motor disturbances such as slowed movement, resting tremors, and change in speech and writing [1]. The absence of dopamine in a person’s organism is also related to depression, memory loss, and disorders in the autonomic nervous system, reducing the living standards of PD’s patients and their relatives [2].

One of the main research areas regarding PD is focused on its diagnosis, mainly in the early stages of the disease. With the technological breakthroughs from recent years, the scientific community came forward with different approaches to solve this problem using various methods, whether they are independent devices or a manner to monitor health variables via sensors. Said solutions aim to facilitate Parkinson’s diagnosis using automatic data processes and analysis via computational intelligence [3].

Albeit the development of these new technologies, in reality, the potential use of such tools to aid in PD’s diagnoses and the cost of developing and implementing them, along with the necessary expertise to maintain their systems operational, have been proved difficult to sustain in some regions, such as rural areas and small villages, where medical care is limited *in loco*.

This work proposes a new electronic device that can be coupled with a normal pen to differentiate the control and PD groups by assessing a simple diadochokinesis paper test, aiding the diagnosis of early stages Parkinson’s. The device is composed of two 3-axis sensors with a total of 6 degrees of freedom (DOF). These sensors send signals, in which a variety of features are extracted, such as pen acceleration and time to complete the task. These features are then analyzed by different Machine Learning (ML) techniques which will be cross-validated to diagnose the patient.

Specifically, this work revolves around using different methods to enhance the overall accuracy of the performance of the diadochokinesis test. The hand maneuverability and speed necessary to perform them need to be modulated and coordinated to produce the required drawings [4]. Researchers have proven that PD patients’ hand motions present different behaviors in regards to range, speed, and time to complete these tests compared to healthy patients [5]. In comparison, a system based in polar coordinates with variable origin was used in the test, which has been proved to have a better performance in the quantitative assessment of the drawings [6]. In this method, the drawing’s origin had a variable starting point, radius, degrees, among other characteristics, which resulted in different parameters for feature extractions, leading to an increase in the accuracy of the test. Moreover, a metric that utilizes normalized speed variability of hand motion between healthy and PD patients was applied since this approach has been noted for having a higher impact in identifying pathological movement patterns [7].

In summary, our contributions are the following:

- We propose a novel and low reproduction cost device, with ease implementation and handling, and a much simpler approach than state-of-the-art.
- Our approach presents advantages in aiding the diagnosis of Parkinson’s disease patients in regions where medical healthcare may be limited. The results can also be analyzed via the Internet, allowing difficult to access areas to receive treatment with the same overall quality as the ones given in metropolitan areas, which is an important feature in the modern medical scenario of online monitoring [8].
- Six new datasets were created from control and test groups, with 20 patients each, and assessed through our proposed device, these being available online (https://github.com/delmiro1/intelipen).

The remainder of this paper is organized as follows. Section 2 presents related work. Section 3 presents the materials and methodology utilized in this work, the overall system, ML techniques used and the resulting dataset proposed. Section 4 presents a description of the tests and the experimental results obtained from the proposed device. Finally, Section 5 states conclusions and future work.

## 2. Related Work

The progressive deterioration of motor function caused by Parkinson’s disease affects diverse functionalities in the human body, mainly speech, posture, and movement. Such deterioration is characterized by muscle stiffness, slow movements, and impaired ability to swiftly move the body on command partially (i.e., bradykinesia) or totally (i.e., akinesia). The main cause of this abnormal condition is the loss of nerve cells in the brain region called substantia nigra, causing reduced formation of dopamine [9].

The diagnosis of PD is generally made using an array of exams, including image signal analysis and motor exams, such as handwriting tests. Lately, these tests have been the center of numerous research works that focus on applying ML techniques to improve and facilitate diagnosis’ results [10]. Reduction in dimensionality, collaborative filtration, and scalable distributed systems are some of the reasons why these techniques can help in the classification task involving PD patients [11].

In a recent study, it was proposed the classification of PD and healthy patients over a database of handwritten tests. This classification would consider the unique characteristics in hand motions of each person’s calligraphy, such as entropy, applied pressure when writing, and spent energy to realize their hand movements. Using an ML algorithm called Support Vector Machines-Radial Basis Function (SVM-RBF), this classification correctly predicted 90% of the subjects’ diagnoses [12].

In Germany, the research led by Dr. Christian Hook resulted in the Biometric Smart Pen (BiSP), a smart biometric pen system for recording and analyzing handwriting, drawing, and gesture movements on a paper pad or free in space [13]. The project initially used for biometric signatures and security applications was later used in Silke A. T. Weber’s study to classify the handwriting patterns of PD’s patients by analyzing their biometric data, for instance, their finger pressure and pen acceleration. These characteristic features extracted from BiSP allowed Silke to correctly classify PD and control group patients with an accuracy above 99.6% [14].

In Brazil, Pereira et al. used a similar approach to Silke’s work but using Convolutional Neural Networks (CNN) to learn features from images produced by handwritten dynamics captured from a computer. The patient realized a common diadochokinesis test where basic shapes are drawn (spiral, meander, and circles) to assess the speed, pressure, tilt, among other features, from each patient, comparing them and using such features to classify control and PD groups [14].

Another example of research in this area was done in 2015 when a study applied Support Vector Machines (SVM) over local field potentials (LFPs) areas in the brain, detected by a deeply implanted device in the basal ganglia. For this study, 83 montages using 15 patients impaired with PD in an advanced stage were made. It used pallidal oscillations as predictive biomarkers for parkinsonian severity, obtaining over 91% accuracy over the test subjects. The strategy implemented in this work consisted of the use of multiple regression normalization techniques to identify numerous time and spatial differences between gaits of healthy and PD patient groups, followed by analyses of the ML efficacy incorrectly classifying the regression differences in each group. The results demonstrated the importance of using ML-based tools to aid in diagnosing PD patients, facilitating the analyses of features, such as spatial-time gait differences, to assess diagnosis correctly [15].

The usage of SVM in conjunction with Bayesian networks also proved effective for differentiating between Parkinson’s disease (PD) and atypical parkinsonian syndromes (APS), a challenging task, especially at early stages. It was evaluated using a database with 87 neuroimages and achieved an accuracy rate of 78% predicting a new, unseen dataset [16].

Following the same subject, Smith et al. employed evolutive algorithms to provide information clinically relevant and objective in order to identify PD in animals and humans in his work “Cartesian Genetic Programming”. The data were obtained via a range of non-invasive procedures that follow the conventional clinical practice, using commercially available sensors. Moreover, this demonstrated that common sensors-based devices can be applied with success in distinguishing PD patients from healthy controls by evaluating their movements and classifying the severity of dyskinesia in these patients [17].

In 2015, a study proposed by Diane J. Cook proved that differences between healthy older adults and adults with Parkinson’s disease not only exist in their activity patterns but can also be automatically recognized using an intelligent system. This system was composed of wearable sensors, in a smart home setting, and a machine learning classifier, which reached an accuracy of 97% in distinguishing both groups, confirming that the sensor-based differences between these groups are statistically significant [18].

Similar results were obtained using a Kinect image and depth sensor for data acquisition and space modulation in a study to analyze the unique characteristics of gait impairment in PD patients. A Bayesian probability classification algorithm was used in features extracted from sensors to predict and classify individuals with Parkinson’s disease and healthy age-matched individuals. The achieved accuracy of the probabilistic classification was 94.1% [19].

More recently in 2019, a software was developed for analyzing images based on drawings assessed by PD and healthy individuals acquired from similar datasets like the one used in this work. It correctly predicted over 96% of the total subjects using ML techniques [20]. Another case of successful use of ML and signal analysis for the detection of PD was done by Jefferson S. Almeida using sustained phonation obtained from speech tasks. The audio recorded from two microphones was processed by multiple classifiers using eighteen different extracted features and achieved between 92.94% and 94.55% accuracy for assessing parkinsonian patients [21]. In the same topic, Laureano Moro-Velázquez obtained 87% accuracy via Kinect change analyses using speech recognition techniques in different application domains [22].

Lastly, G. Singh and L. Samavedham [23] proposed an innovative project based in Kohonen Self-Organizing Map (KSOM) and least squares support vector machine (LS-SVM) for monitoring the progression of neurodegenerative diseases and to aid in its individual-level clinical diagnosis. This approach achieved over up to 99% accuracy for differential diagnosis of Parkinson’s disease using 831 T1-weighted magnetic resonance imaging (MRI) from Parkinson’s Progression Markers Initiative (PPMI) database.

As aforementioned in these examples, the use of signal-based methods, such as electronic sensors and ML techniques has proved to be a reliable procedure in identifying biomarkers, aiding the diagnosis of neurodegenerative disease patients, and demonstrating the applicability of this methodology for diagnosing PD subjects [3,24,25,26,27,28,29,30,31,32,33,34].

## 3. Materials and Methods

The procedure utilized here was divided into several steps. Initially, the development of an electronic device capable of collecting information from handwriting tests, useful enough to correctly classify PD patients from healthy ones. This information was gathered as signals from the device’s sensors, which was then processed, extracting a series of features, leading to the final step in the system, the analyses of features by ML, and final assessment of the patient’s diagnosis. All these steps will be further explained throughout this section.

### 3.1. The Intelligent Pen Prototype (an Electronic Device)

**Figure 1:**
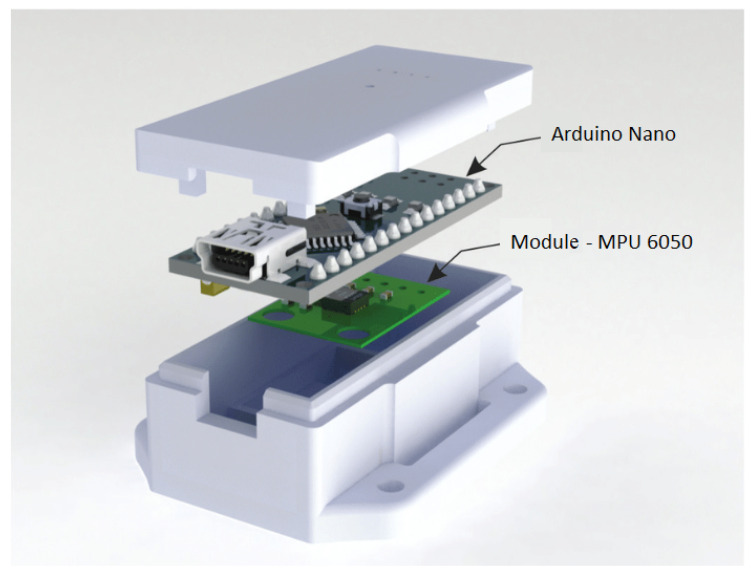
The intelligent pen prototype design.

The electronic device here developed has an Arduino based implementation (Arduino Nano R3), with an MPU 6050 module as shown in Figure 1. This module has two 3-axis sensors, i.e., an accelerometer and a gyroscope, resulting in total six degrees of freedom (DOF). These sensors are responsible for capturing the signals from the handwritten test, allowing for the extraction of features.

**Figure 2:**
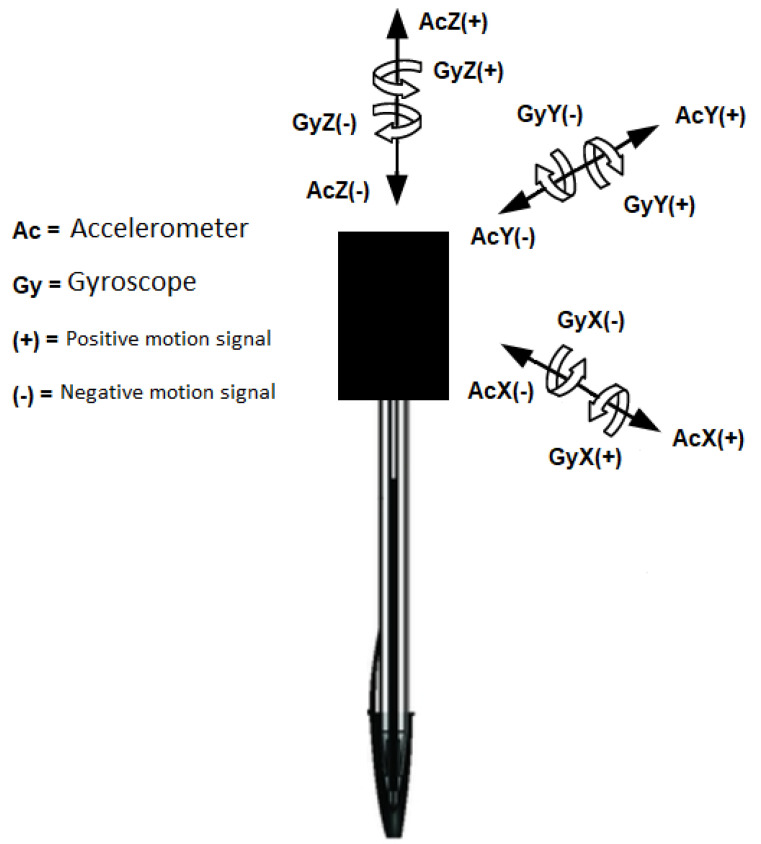
Sensors positioning and degrees of freedom (DOF) of the electronic device.

The Arduino can be programmed on its native Integrated Development Environment (IDE) in C or C++ languages. This work used an open-source code for detecting, measuring, and collecting the signal data from the sensors. The accelerometer sensor can detect linear motions and, for this reason, was employed to detect and measure the features related to entropy and energy spent in the handwritten task, such as the applied pressure and acceleration from the pen. Likewise, the gyroscope was utilized in detecting the angular motions, from which hand stiffness and tremors could be perceived. Both sensors range from negative to positive values and can detect the three spatial orientations along their *X*, *Y* and *Z* axis as shown in Figure 2, allowing for tracking the patient’s hand accurately continuously.

### 3.2. Handwritten Tests and Dataset

There are numerous types of tests focused on PD diagnosis, mostly based on signs and biomedical imaging, such as electroencephalogram, computed tomography, magnetic resonance, and speech analysis [35]. Recently, the use of biometric tests has become increasingly popular and promising for providing more effective clinical information to be analyzed in conjunction with multi-sensors and intelligent systems tools.

**Figure 3:**
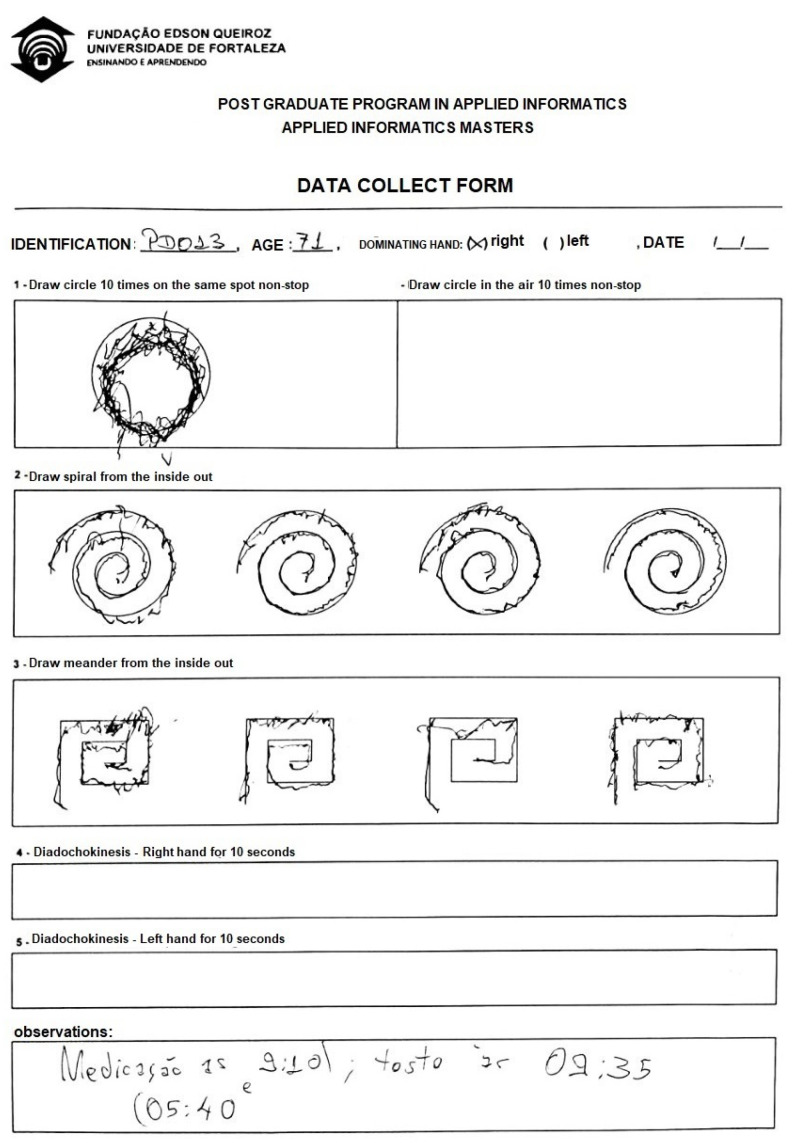
An example of the test applied.

The handwritten test applied in this work was based on Clayton Pereira’s research about biometric sensors for aiding in the detection of PD using ML, which proved to be effective in said task. The test utilized in such work, i.e., the HandPD Dataset, consists of six different activities as shown in Figure 3, which focus in quantifying the normal motor activity in a healthy individual, as well as the motor dysfunction of PD patients. The first four tasks consists in the drawing of different shapes, such as meanders, spirals, and circles, with the two final tasks being the diadochokinesis tests, in which the individual performs hand-wrist movements with straight arms, for both arms.

These tests took place in the Hospital Geral de Fortaleza (HGF) over three months. For the experiment, 40 individuals clinically diagnosed by a doctor specialist, to ensure the validity of the tests, from different ages ranging from 55 to 88 years old, have been selected and divided in two groups: (i) the first one containing 20 healthy individuals, named HC group, with 8 male subjects and 12 female; (ii) the second group contains 20 patients affected with PD, named PD group, having 12 male subjects and 8 female ones. The patients in the PD group had a moderate Parkinson’s Disease, with an average UPDRS, and Hoehn and Yahr scores of 28.5 ± 5.5 and 2.2 ± 0.2, respectively. They were asked to fulfill the *HandPD* form in the following order using the intelligent pen:

- Draw circles on the form
- Draw circles in the air
- Draw spirals on the form
- Draw meanders on the form
- Right-hand diadochokinesis
- Left-hand diadochokinesis

Each task on the form was realized four times, while tasks in the air were done ten times, thus ensuring evenly average results across the testings.

**Figure 4:**
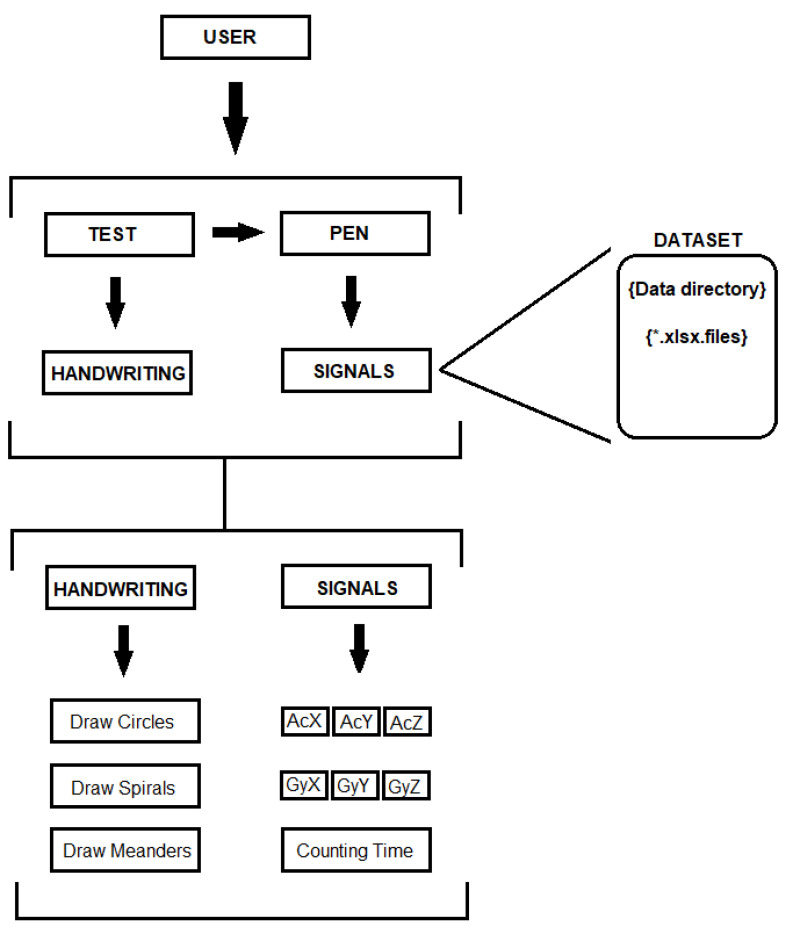
The architecture of the modeled system.

Meanwhile, a computer connected to the electronic device gathered the data from the sensor’s signals at a rate of 1 millisecond each for both accelerometer and gyroscope. This data was automatically parsed to an Excel spreadsheet as columns for each of the three-axis from both sensors, such as AcX, AcY, and AcZ for the accelerometer signals, and GyX, GyY, and GyZ for the gyroscope signals. Furthermore, the final data was grouped and organized by each sensor, and both combined, resulting in a new dataset composed of all six tasks in three groups of settings: accelerometer only, gyroscope only, and using both combined. The complete system architecture is shown in Figure 4.

**Figure 5:**
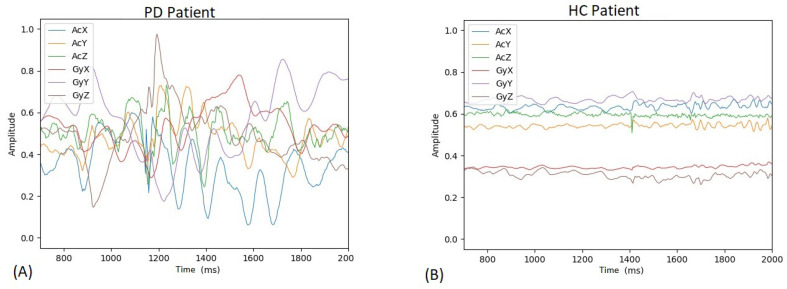
Example of the signal acquired from the sensors for (**A**) Parkinson’s disease (PD) individual and (**B**) healthy ones.

Patients with PD often presented a movement disorder dysfunction characterized by jolty motions or spasms, whereas healthy individuals (HC) demonstrated a standardized pattern of movements, as shown in Figure 5.

This group of PD patients was chosen to exclude other Parkinsonism causes by clinical criteria, since most causes of atypical parkinsonism present additional symptoms as the disease progresses. Most patients had the disease for more than ten years and were tested using their regular medication for Parkinson’s treatment. Nonetheless, they were considered valid for the purposes of this study, given the difficulty in finding a sufficiently large number of patients willing to take the tests. All were taking levodopa, and tests were performed with patients in the “on” phase.

Meanwhile, the tests from individuals under more aggressive treatment, in which the results were redundant due to intense medications, have been disregarded.

### 3.3. Data Pre-Processing and Ml

The data collected from the tests were compiled and computed using 13 statistical features, often used in activity recognition due to its superior performance when utilized in conjunction with ML computing [11], as presented in Table 1.

They were extracted from the fulfilled tasks, independently for both sensors in each of three settings (accelerometer only, gyroscope only and both simultaneously), resulting in 66 total features. The absolute values were not considered since the starting values for the sensors will always differ based on multiple factors, such as each person’s handedness and how they grip the pen. Instead, we used the ratio in which the values from the sensors varied over time since the ML will consider their offset to determine the patient’s effort (how “hard” was for them) to perform a task. The data was normalized to prevent issues when using different ML algorithms, such as different ranges for features, unbalanced samples in the dataset, and overfitting the data. This normalization consists of several steps, such as: eliminating duplicate data, resolving conflicting data, and formatting it, converting into a format that allows further processing [36].

**Figure 6:**
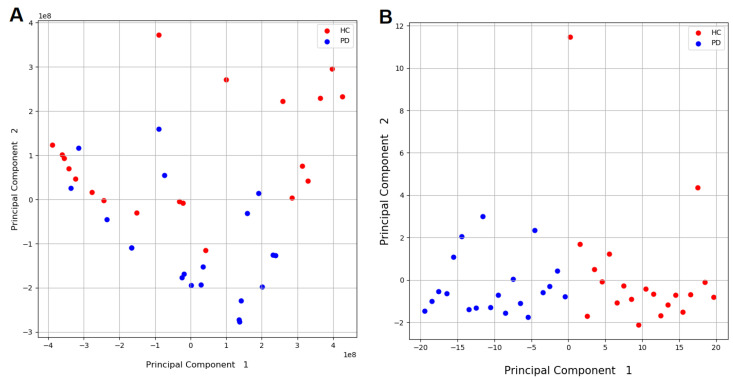
Example of principal component analysis (PCA) before (**A**) and after (**B**) normalization, demonstrating the clustering between healthy individuals (HC) and PD individuals as perceived by the system.

Moreover, principal component analysis (PCA) is applied to the new data window containing the 66 samples for each task, arranged into a vector with corresponding entries for each sensor axes (X, Y, and Z). This yields the projection matrix, with all data vectors stacked and disregarding class labels, which is finally used in the testing phase to project the single data frames. The objective of this step is to reduce the data, preserving the essential parts in it that have more variation and removing non-essential parts with fewer variations, optimizing the classification process for the ML [37], as shown in Figure 6.

Furthermore, six different classifiers methods were used for solving the classification task in this work:

- Linear Discriminant Analysis (LDA)
- Logistic Regression (LR)
- Classification and regression Trees (CART)
- K-Nearest Neighbors (KNN)
- Support Vector Machines (SVM)
- Naive Bayes (NB)

These classifiers have been commonly applied in the categorization of extracted features in other related works in this area due to their simplicity and overall performance in similar tasks and by combining their results, and the possibility to reduce variance in final classifications [38]. Due to the sizable data available in this study, a *k-fold cross-validation* was used to split between training and testing for all ML algorithms. Since this method evaluates the performance of any ML model used in every iteration possible from the original dataset, it ensures that all the data will be used for training and testing at random, reducing the bias of the classification model.

Additionally, in order to assess these models, four common evaluation methods were calculated:

- Accuracy: the number of correct predictions to the total number of input samples ratio;
- Precision: the ratio between the number of correct positive results and all positive results (i.e., false positives) predicted by the classifier;
- Recall: the ratio between all correctly positive results assessed and the number of all relevant samples;
- F1-Score: the harmonic average between precision and recall. The range for the F1 score is 0 to 1 and it represents how accurate the classifier was.

These methods are commonly employed in ML related works since they can significantly evaluate the overall performance of classification tasks models. Moreover, classical algorithms are also advantageous in this study since they are easier to implement, have space complex solutions, have high-speed training, support linear and non-linear solutions, and perform overall better when the training data has a smaller value, like the one used in this work.

## 4. Experimentation and Results

**Figure 7:**
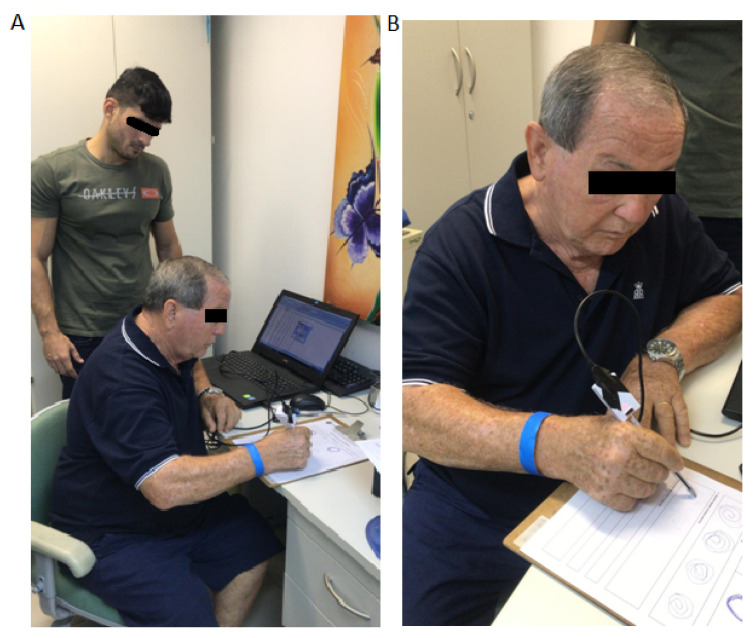
In (**A**) the system setup for the test and in (**B**) a patient using the intelligent pen.

In this section, the results are presented. The electronic device developed was tested using three different settings, accelerometer only, gyroscope only, and both combined, to assert the optimal configuration in which the device would have better performance and higher overall accuracy. In Figure 7, the setup for testing and the intelligent pen is shown.

A doctor supervised the patients, and the system responsible for collecting all data was also monitored at all times to guarantee the validity of the tests. Furthermore, the results were analyzed individually, as new datasets, for each performed task:

- Circle dataset
- Air-Circle dataset
- Spiral dataset
- Meander dataset
- Diadochokinesis dataset

All datasets were split into two categories, i.e., training and testing, being 75% and 25% for the first and second, respectively. The results from these tests are now presented.

### 4.1. Circle Dataset

Initially, in the task of drawing the Circle, it was found that the CART classifier, when using accelerometer and gyroscope together, obtained the best results:

- Accuracy: 100% for both groups
- Precision: 100% for both groups
- Recall: 100% for both groups
- F1-Score: 100% for both groups

**Figure 8:**
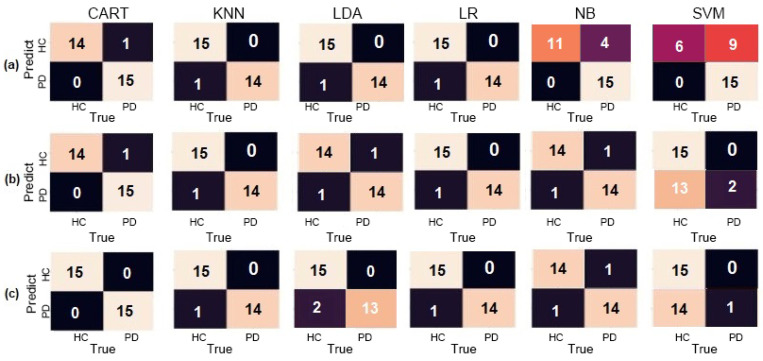
Confusion matrices for the Circle dataset for (**a**) Accelerometer only, (**b**) Gyroscope only, and (**c**) both combined.

LDA, LR, KNN, and NB classifiers had similar results, achieving over 90% accuracy in different configurations. SVM had the worst results, with the lowest accuracy of 53.33% and F1-Score of 12.50% when using the accelerometer to identify PD individuals. These results are better demonstrated using a confusion matrix, as shown in Figure 8.

**Figure 9:**
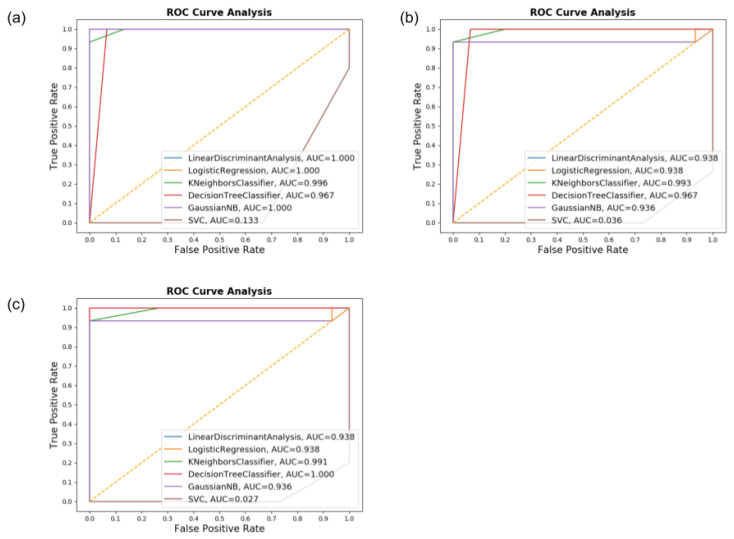
The Receiver Operating Characteristic (ROC) graph and Area Under the Curve (AUC) values for the Circle dataset for (**a**) Accelerometer only, (**b**) Gyroscope only, and (**c**) both.

In Figure 9, the Receiver Operating Characteristic (ROC) is shown. This provides the Area Under the Curve (AUC), which is a metric to evaluate all the classifiers’ output quality. It is visible in the graph that the results were very similar between multiples classifiers, with overall AUC above 0.9. This result proves the efficiency of the classifiers in this specific task since the low rate of false positives and false negatives means a correctly assessed patient using the ML.

According to the patients who performed the task, this proved to be one of the easiest to accomplish with its values above the average when compared with others in this work, corroborating the statement.

### 4.2. Air-Circle Dataset

In the Air-Circle test, the LR classifier successfully achieved 100% in its assessments in all configurations. The scores were:

- Accuracy: 100% for both groups
- Precision: 100% for both groups
- Recall: 100% for both groups
- F1-Score: 100% for both groups

**Figure 10:**
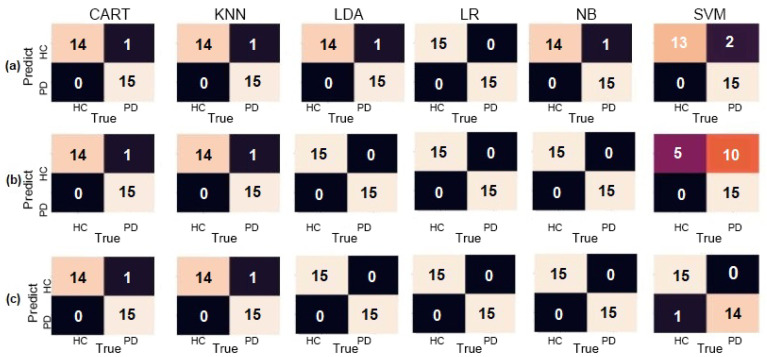
Confusion matrices for the Air-Circle dataset for (**a**) Accelerometer only, (**b**) Gyroscope only, and (**c**) both combined.

However, LDA, KNN, CART, and NB also obtained exceptional results by correctly assessing all parkinsonian patients in all three configurations, presenting no false positives. SVM presented the worst results with 66.67% accuracy and F1-Score of 75% for patients with PD when using gyroscope only. These results with the confusion matrix are observable in Figure 10, showing mostly classifiers obtaining maximum accuracy.

The data from PD and HC individuals in the Air-Circle task presented a higher variance, seemingly accomplishing better results, presumably due to the difficulty in sustaining their hand in motion necessary to complete the task for prolonged periods of time when performed by PD patients in contrast to healthy individuals.

**Figure 11:**
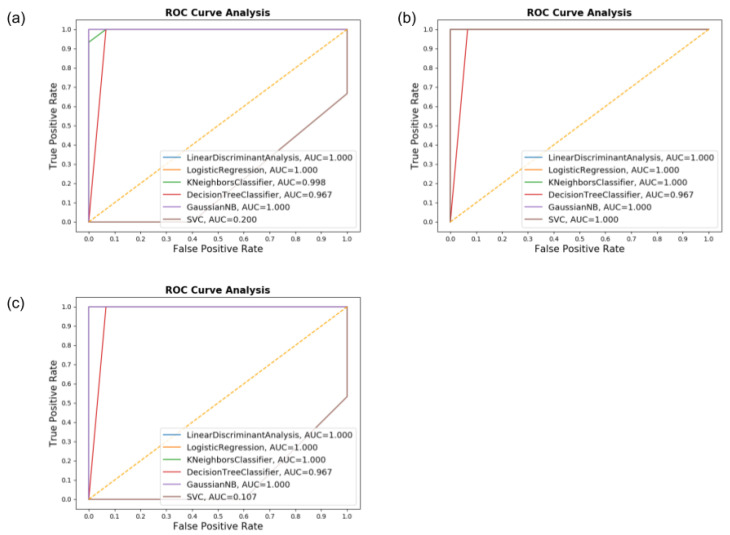
The ROC graph and AUC values for the Air-Circle dataset for (**a**) Accelerometer only, (**b**) Gyroscope only, and (**c**) both.

The ROC and AUC graphs presented in Figure 11 demonstrates the results. The performance of all classifiers when using both accelerometer and gyroscope demonstrated to be of high efficiency. Still, when using gyroscope only, the results presented a slight decrease in the overall quantity of correctly assessed patients, mostly from the HC group, with the SVM classifier displaying a steep drop. Despite this, most classifiers performed well with AUC values above 0.9.

### 4.3. Spiral Dataset

The Spiral dataset presented the worst results between all tasks. From observation, it is possible to conclude that this task showed a higher presence of “noisy” data from PD and HC individuals alike. While it is not possible to state the reason for such a problem, we believed it is due to the sensors’ inadequacy to the spiral shape. Regardless, the CART classifier, using accelerometer only, still presented a sufficiently accurate performance:

- Accuracy: 80% for both groups
- Precision: 90.90% for PD and 73.68% for HC
- Recall: 66.67% for PD and 93.33% for HC
- F1-Score: 76.92% for PD and 82.23% for HC

**Figure 12:**
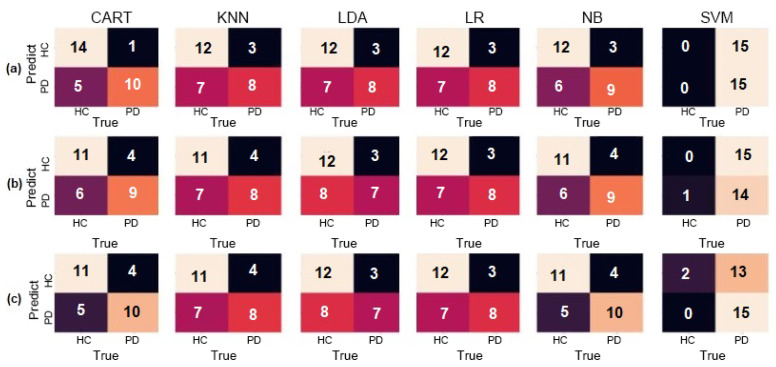
Confusion matrices for the Spiral dataset for (**a**) Accelerometer only, (**b**) Gyroscope only, and (**c**) both combined.

While SVM had the worse results when using both sensors at once, with an average accuracy of 46.67% and an F1-Score of 23.53% when trying to predict PD patients correctly. Figure 12 displays the confusion matrix for the spiral dataset in which it is possible to notice a presence of false positives and false negatives above average in most classifiers.

**Figure 13:**
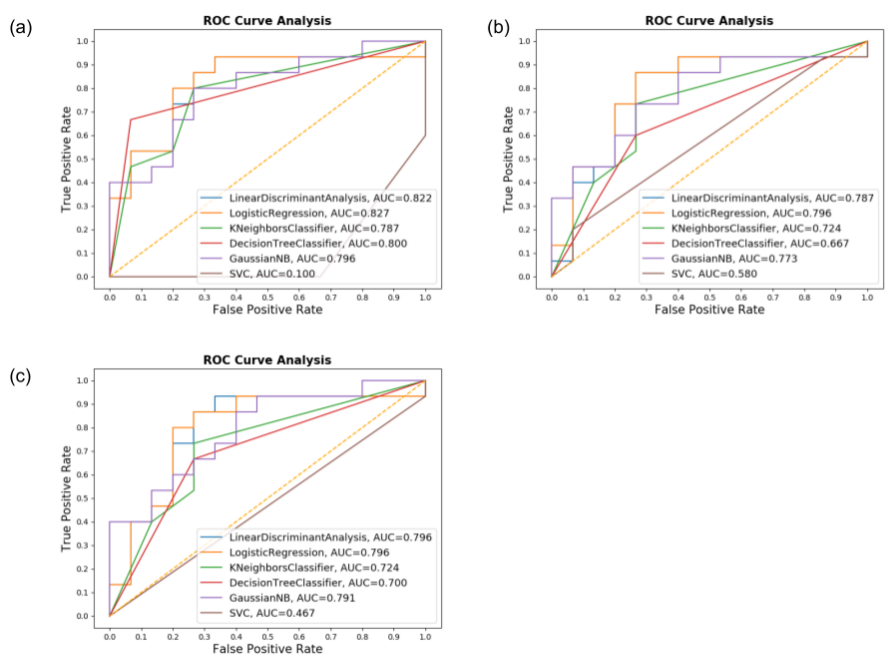
The ROC graph and AUC values for the Spiral dataset for (**a**) Accelerometer only, (**b**) Gyroscope only, and (**c**) both.

These results can be seen better with the ROC graph in Figure 13. The AUC averaged below 0.8 in this task, being perceived with a high amount of false positives and negatives, mostly when assessing PD patients. Nevertheless, the configuration using the accelerometer was only performed with values of AUC above 0.8 when paired with CART, proving to be the best setting for this task.

### 4.4. Meander Dataset

When assessing the Meander dataset, the LDA and LR algorithms achieved the same results when using only the accelerometer:

- Accuracy: 90% for both groups
- Precision: 100% for PD and 83.33% for HC
- Recall: 80% for PD and 100% for HC
- F1-Score: 88.89% for PD and 90.91% for HC

Contrastingly, the SVM classifier obtained only 56.67% accuracy and F1-Score of 23.53% when trying to identify PD individuals when using gyroscope only, which was the worst result in this. The meander test results demonstrated to be the most difficult task for PD patients to carry out due to the nature of hand motion necessary to accomplish the test.

**Figure 14:**
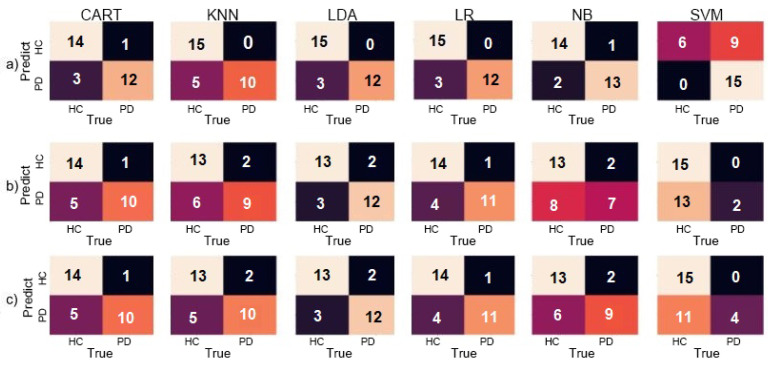
Confusion matrices for the Meander dataset for (**a**) Accelerometer only, (**b**) Gyroscope only, and (**c**) both combined.

In Figure 14, the results are perceptible, since there is a significantly higher number of false positives than negatives in most classifiers, corroborating with the previous statement.

The Meander dataset also presented a higher average time necessary to complete in comparison with the other tasks. It presented inferior results when compared with a different task due to the time to completion for PD individuals, which directly impacts their performance since their hands tend to tire faster and easier than healthy individuals.

**Figure 15:**
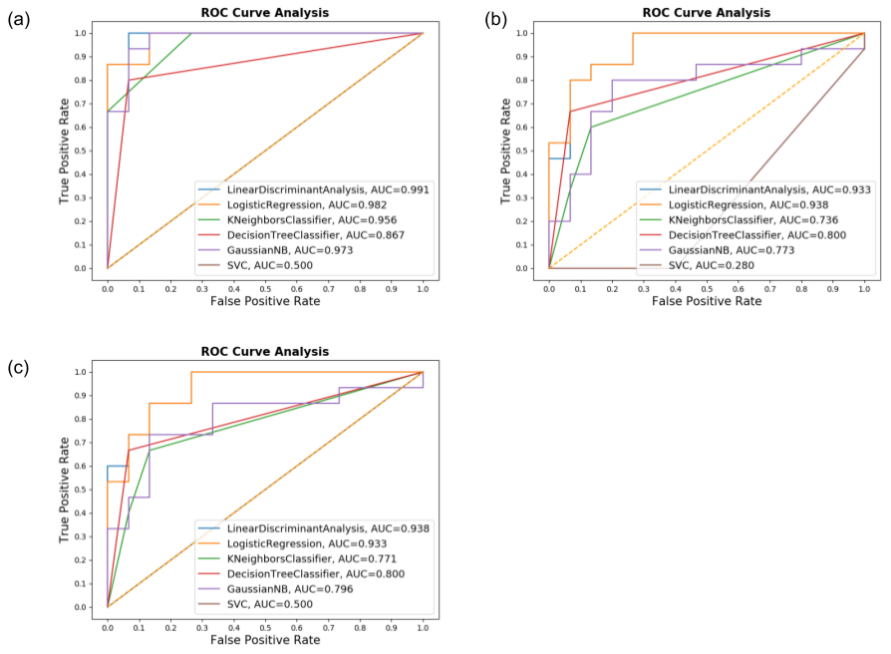
The ROC graph and AUC values for the Meander dataset for (**a**) Accelerometer only, (**b**) Gyroscope only, and (**c**) both.

Nevertheless, most classifiers performed exceptionally well in all configurations, but mainly using the accelerometer only, which presented an average AUC above 0.99 as shown in Figure 15.

### 4.5. Diadochokinesis Dataset

The Diadochokinesis test was made two times, one for each hand and presented excellent outcomes in both, with the best results overall been LR and CART for both hands:

- Accuracy: 100% for both groups
- Precision: 100% for both groups
- Recall: 100% for both groups
- F1-Score: 100% for both groups

**Figure 16:**
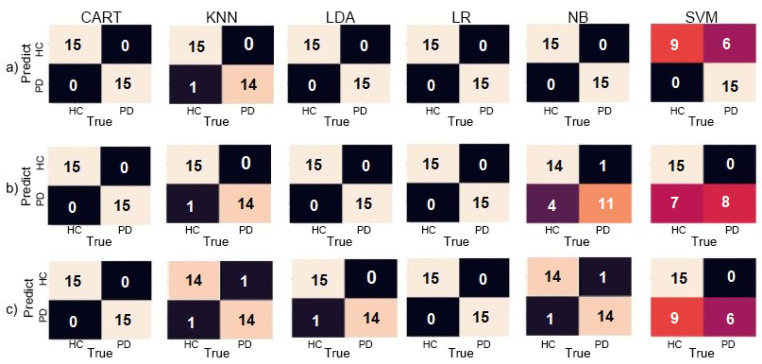
Confusion matrices for the Diadochokinesis dataset for (**a**) Accelerometer only, (**b**) Gyroscope only, and (**c**) both combined.

Furthermore, using the gyroscope only, the SVM had the worse performance with an overall accuracy of 66.67% and, when assessing PD individuals, F1-Score of 50%. These results are shown in Figure 16, with its confusion matrix, demonstrating that this task presented almost no false negatives and positives in multiple classifiers.

Since the test was made multiple times with both hands and averaged for its results, it is believed that the repetitions have significantly impacted the tasks’ overall performance.

**Figure 17:**
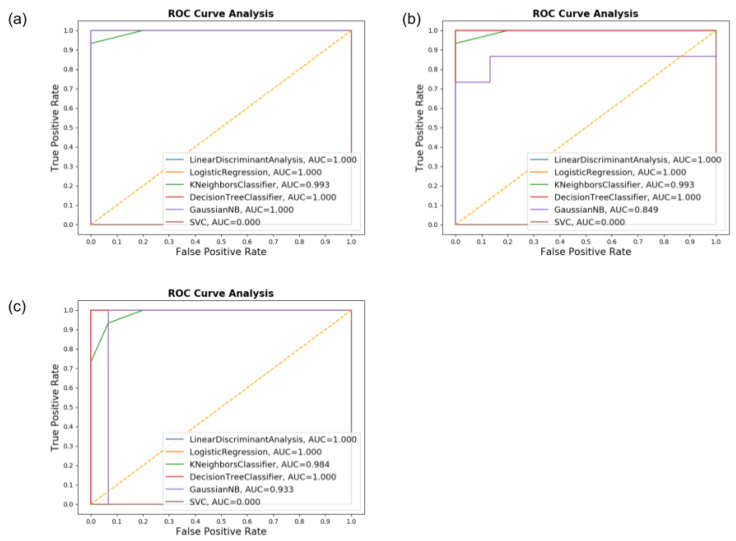
The ROC graph and AUC values for the Diadochokinesis dataset for (**a**) Accelerometer only, (**b**) Gyroscope only, and (**c**) both.

The method in which the test is applied accounts also for the optimal results since the test focuses on quick hand movements and alternate motions, which are known difficulties for PD patients overcome due to movement impairment. These results can be seen with the ROC graph and its AUC values, as shown in Figure 17.

The LR and CART algorithm presented an AUC of 1.0 in all configurations, meaning no false positive or negative for these two classifiers, specifically, confirming the validity of the classification assessments when performed with those settings.

## 5. Conclusions and Future Work

This paper proposed an electronic device capable of detecting standards and deviations on individuals’ handwritings, such as tremors, orientation, and speed. This device being attachable to a common pen can be employed as an auxiliary tool to aid in the detection and diagnosis of Parkinson’s disease. The features extracted from the device were analyzed using ML techniques over a 10-fold cross-validation procedure and 25% of the dataset for training, with the remaining dataset being used for testing purposes. This method proved to be efficient in distinguishing a health control group from a PD’s one with overall accuracy close to 100% over multiple classifiers. However, since most studies presented in this work used different methodologies to accomplish the task determined, such as using image analysis instead of dynamic handwriting analysis, the database being new and its use unprecedented, it was not possible to compare these results with the current work in the state-of-the-art literature or with other movement disorders. For the latter, we also believe that it is important to test this device in other hyperkinetic conditions in future studies, since a differential diagnosis must be made for these conditions.

This work compiled six new datasets from six different tasks over 40 different patients. Each one was composed of multiple signals from the intelligent pen sensors, hoping to aid future researchers in Parkinson’s disease diagnoses.

The *HandPD* Dataset was used to learn valuable and essential information about the limitations of the mentioned device. Such limitations, i.e., the sensor’s disposition, were responsible for ‘noisy’ data and higher deviation in specific tasks than others. Yet, even with data quality lower than expected, the overall results still surpassed the anticipated. This information proved the device viability and effectiveness in aiding the classification of healthy and parkinsonian groups using an affordable and handier solution than others offered in the market, mainly for faraway places and undeveloped towns. This being the main objective of our work. The low-cost electronics and easier setup for acquiring and assessing the data also contribute to the goals mentioned above.

As future works, the device can be further developed to process the diagnoses and classification by its own, using built-in hardware, as well as allowing the interconnection between multiple devices and the internet for IoT related purposes. This would make an even handier tool for PD’s diagnoses in even more remote and isolated areas. Besides, using better sensors would allow eliminating present errors and improving the overall efficiency of the device. Alongside the electronic improvements, the entirety of this study could benefit from a larger number of patients, during an extended period of time and accompanied by specialists in the area, which could significantly improve the test results and perform a accurate clinical validation of the database and the proposed device.

Finally, with the breakthrough of new algorithms and techniques, focusing on artificial intelligence and classifications, an approach more efficient for aiding in solving the classification problem for PD, as well as other movement disorders, using the device developed in this work may be possible.

## Figures and Tables

**Table 1:** Computed statistical features and their descriptions.

Features	Description
Min	Minimum signal value
Max	Maximum signal value
Median	Median signal value
Mean	The average value of the signal
Root Mean Square	The quadratic mean value of the signal
Variance	Square of the standard deviation
Standard Deviation	Mean deviation of the signal compared to the average
Kurtosis	The degree of peakedness of the sensor signal distribution
Skewness	The degree of asymmetry of the sensor signal distribution
Mode	Most frequent numbers in the signal
TrimMean	Trimmed mean of the signal in the window
